# Supplementary material for: Whole-Genome Sequencing Improves the Diagnosis of DFNB1 Monoallelic Patients
Source: Genes (Basel). 2021 Aug 19;12(8):1267. doi: 10.3390/genes12081267 (PMC8391926; doi:10.3390/genes12081267)
Supplement: Supplementary file 1 [file genes-12-01267-s001.zip › genes-1347437-supplementary.pdf]

Supplementary material

Table S1: 215 deafness genes screened

ABHD12	CDH23	DIABLO	G1PC3	ILDR1	MITF	NLRP3	PRKCB	SLC52A3	TNC
ACTG1	CEACAMI6	DIAPH1	G4TA3	JAG1	MPZL2	NPTN	PRORP	SLC9A1	TPRN
ADCY1	CEP78	DIAPH3	GATA2	KARS	MRPS7	OPA1	PRPS1	SLC9A3R1	TR1OBP
AIFM1	CHD7	DMXL2	GGPS1	KCNE1	MSRB3	OSBPL2	PTPN11	SMPX	TRPP3
ALMS1	CLDNI4	DNFB59	GJB1	KCNJ10	MT -TH	OTOA	PTPRQ	SNAI2	TSHZ1
ANKRD11	CLIC5	DSPP	GJB2	KCNQ4	MT-RNR1	OTOF	RAF1	SOX10	TSPEAR
ATP1A3	CLPP	EDN3	GJB3	KCNQI	MT-TE	OTOG	RDX	SPTBN5	TUBB4B
ATP2B2	CLRN1	EDNRB	GJB4	KDM6A	MT-TF	OTOGL	ROR1	STRC	TWNK
ATP6V1B1	CLRN2	ELMOD3	GJB6	KIAA1199	MT-TI	PAX3	RPGR	SYNE4	TWNL4A
ATP6V1B2	COCH	EPS8	GPR98	KITLG	MT-TK	PCDHI5	S1PR2	TBC1D24	TYR
BCS1L	COL11A1	EPS8L2	GPRASP2	KMT2D	MT-TL1	PDXDCI	SERPINB6	TBLIXR1	USH1C
BDP1	COL11A2	ERAL1	GPSM2	LHFPL5	MT-TS1	PDZD7	SIX1	TECTA	USH1G
BSND	COL2A1	ESPN	GRHL2	LMX1A	MYH14	PEX1	SL1TRK6	TIMM8A	USH2A
C0L9A2	COL4A5	ESRP1	GRHLI	LONPl	MYH7B	PEX26	SLC17A8	TJP2	VEZT
C1B2	COL4A6	ESRRB	GRXCR1	LOXHD1	MYH9	PEX6	SLC19A2	TMC1	VPS13B

C1SD2	COL9A1	EYA1	GRXCR2	LRTOMT	MYO15A	PEX7	SLC22A4	TMC2	WBP2
CABP2	COL9A3	EYA4	HARS	MAF	MYO1A	PHYH	SLC26A4	TMEM126A	WFS1
CACNA1D	CRYM	FAM65B	HARS2	MARVELD2	MYO3A	PLCB3	SLC26A5	TMEM132E	
CCDC50	DAMT1	FDXR	HGF	MCM2	MYO6	PMP22	SLC29A3	TMIE	
CD151	DCDC2	FGF3	HOMER2	MCOLN3	MYO7A	PNPTI	SLC44A4	TMPRSS1	
CD164	DFNA5	FGFR3	HSD17B4	MET	NARS2	POU3F4	SLC4A11	TMPRSS3	
CDCI4A	DFNB31	FOXII	IARS2	MIR96	NLRP12	POU4F3	SLC52A2	TMTC2	

Table S2: PCR primer sequences used for cloning into the luciferase reporter construct (5' - 3')

			**genomic position chr13 (hg38)**			**distance from the *GJB2* transcriptional start site (kb)**	
PGJB2	F	CGATCTAAGTAAGCTTCGGTGAATTTAAAACGTTTGGTGGC	20,192,394	20,193,934			
	R	CCGGAATGCCAAGCTTCCGCAGAATCCTATCAGTTTCC					
C3	F	ATAAGGATCCGTCGACCTGCTTCGGCCTCCAAATAG	20,419,403	20,420,445		-221.8	-222.9
	R	AAGGGCATCGGTCGACCTTCTTCTGGAGTCTTTTCTGG					

Table S3: PCR primer sequences used for mutagenesis (5' - 3')

			**genomic position chr13 (hg38)**	
PromGJB2_rs1425012952	F	ccgcggcgcggccccctcc	20,193,013	20,193,031

PromGJB2_rs1425012952	R	ggagggggccgcgccgcgg		
